# Acute and chronic effects of exercise on mRNA expression in the skeletal muscle of two mouse models of peripheral artery disease

**DOI:** 10.1371/journal.pone.0182456

**Published:** 2017-08-03

**Authors:** Hiroki Nagase, Shuhei Yao, Shota Ikeda

**Affiliations:** 1 Cardiovascular and Metabolic Drug Discovery Unit, Pharmaceutical Research Division, Takeda Pharmaceutical Company Limited, Fujisawa, Japan; 2 Integrated Technology Research Laboratories, Pharmaceutical Research Division, Takeda Pharmaceutical Company Limited, Fujisawa, Japan; University of Minnesota Medical Center, UNITED STATES

## Abstract

Endurance exercise improves walking performance in patients with peripheral artery disease (PAD), which is characterized by skeletal muscle dysfunction caused by lower extremity ischemia. Although transcriptional analyses of exercise-induced changes in normal animals and healthy volunteers have been reported, no detailed study has explored exercise-induced alterations in gene expression in PAD animal models. Here, we determined the acute and chronic effects of exercise on mRNA expression in the skeletal muscles of two mouse models of PAD. Three particular gene categories were investigated: known exercise-responsive genes (*Pgc1a*, *Il6*, *Nr4a1*, *Nr4a2*, and *Nr4a3*); myogenic and muscle regeneration-related genes (*Myf5*, *Myogenin*, *Myomaker*, and *Myh3*); and *Gpr56* and its ligand *Col3a1*. PAD was induced by bilateral femoral artery ligation in normal C57BL/6 and diabetic KK-*A*^*y*^ mice. From 1 week after surgery, repetitive twice-weekly 30-min treadmill endurance exercise sessions were applied. Altered mRNA expression in the soleus muscles was measured in both the acute and chronic phases. In the acute phase, transcript levels of exercise-inducible genes showed significant increases in both C57BL/6 and diabetic KK-*A*^*y*^ PAD mice; levels of regeneration-related genes showed little alteration, and those of *Gpr56* increased immediately and significantly after exercise in both models. In the chronic phase, transcript levels of *Pgc1a*, *Myf5*, *Myogenin*, *Myomaker*, *Myh3*, *Gpr56*, and *Col3a1* were upregulated significantly in sedentary C57BL/6 PAD mice compared with that in sham-operated mice. Exercise training inhibited the upregulation of *Col3a1*, *Myf5*, and *Myogenin* significantly. In KK-*A*^*y*^ PAD mice, only *Gpr56* mRNA levels increased significantly compared with those in sham-operated mice. RNA sequence analysis revealed 33 and 166 differentially upregulated, and 363 and 99 downregulated, genes after exercise training in C57BL/6 PAD and KK-*A*^*y*^ PAD mice, respectively. In summary, we detected significant alterations of skeletal muscle genes after exercise in PAD mouse models and characterized their expression patterns.

## Introduction

Peripheral artery disease (PAD) is a disorder attributed to atherosclerotic occlusion in the lower extremities. PAD is characterized by skeletal muscular pain and dysfunction caused by insufficient blood supply [1]. A large population of patients with PAD is affected with concomitant coronary and cerebrovascular diseases, resulting in high mortality and morbidity in developed and developing nations [2]. The most common symptom of PAD is intermittent claudication (IC), characterized by exercise-induced discomfort that is relieved by rest [3]. The primary goal for patients with PAD and IC is to improve exercise performance, daily functional activity, and quality of life. Recent evidence revealed that exercise training has beneficial effects in extending the walking distance for patients with PAD and IC [4]. A randomized trial of exercise training added to optimal medical therapy, including cilostazol, demonstrated a clear additive benefit, even superior to that of aortoiliac revascularization [5]. Exercise training is considered to affect several machineries associated with clinical benefits, including alteration in skeletal muscle metabolism, conditioning underlying diseases, and improving endothelial function [5–8]; however the detailed mechanisms remain unknown. A better understanding of the molecular mechanism underlying the exercise-induced benefits is thus highly desirable.

There is accumulating evidence that exercise training can induce significant alterations in the expression levels of several genes in skeletal muscles; this effect is considered one of the most important underlying molecular mechanisms of exercise-induced benefits. For instance, interleukin-6 (IL-6), which is locally produced in skeletal muscles in response to exercise [9], is known to increase glucose availability and activate muscle satellite cells, contributing to muscle hypertrophy and regeneration [10, 11, 12]. NR4As (*NR4a1*, *NR4a2*, and *NR4a3*) are other genes that are reported to be induced by endurance exercise in human skeletal muscle [13] and are considered to play a role in exercise-induced benefits through improved muscle energy metabolism [14]. These considerations are largely based on the reported data from experiments on normal animals and healthy volunteers. To the best of our knowledge, to date, no detailed studies have described exercise-induced alterations in gene expression in PAD animal models and patients with PAD.

Bilateral ligation of the femoral arteries (FAL) is one of the most common methods to induce PAD in animal studies. Many investigators [15], including ourselves [16], have reported that several key characteristics of PAD, including muscle atrophy, apoptosis, fiber type switching, altered myosin heavy-chain expression, and muscle fiber denervation, can be induced in mice and rats using this surgery. In agreement with the fact that diabetes is an important risk factor for PAD patients [17], diabetic animals such as KK-*A*^*y*^ mice have been reported to show more severe disease phenotypes [18,19].

Recently, White et al. reported that G protein-coupled receptor 56 (GPR56) might play an important role in muscle hypertrophy associated with resistance/loading-type exercise [20]. A murine model of overload-induced muscle hypertrophy is associated with increased expression of both *Gpr56* and its ligand, collagen type III, whereas genetic ablation of *Gpr56* expression attenuated overload-induced muscle hypertrophy and associated anabolic signaling. In addition, *GPR56* expression was induced in humans by resistance exercise [20]. Although these new findings suggest GPR56 could be an interesting molecular target to mimic the molecular signaling related to exercise-induced clinical benefits, the expression profiles of GPR56 have not been reported in PAD animal models and patients with PAD.

The aim of the present study was to determine the acute and chronic effects of exercise on mRNA expression in the soleus muscles of two mouse models of PAD: normal C57BL/6 mice and diabetic KK-*A*^*y*^ mice with FAL. In particular, we focused on three categories of genes: 1) known exercise-responsive genes (*Pgc1a*, *Il6*, *Nr4a1*, *Nr4a2* and *Nr4a3*); 2) myogenic and muscle regeneration-related genes (*Myf5*, *Myogenin*, *Myomaker* and *Myh3*); and 3) *Gpr56* and its ligand *Col3a1*. In the present study, we also investigated whether diabetes could induce exercise-induced transcriptional alterations, by comparing the data obtained from the two PAD mouse models.

## Materials and methods

### Animals

Male C57BL/6 mice and KK-*A*^*y*^/Ta mice (8 weeks old) were purchased from CLEA Japan (Tokyo, Japan). All animals were maintained on a laboratory chow diet (CE-2, CLEA Japan) and allowed free access to water and food before and during the experiments. The care and use of the animals and the experimental protocols used in this research were approved by the Institutional Animal Care and Use Committee of Takeda Pharmaceutical Co., Ltd. (Approval code: 00005751)

### Bilateral femoral artery ligation (FAL)

C57BL/6 and KK-*A*^*y*^ mice underwent FAL at 12 and 10 weeks of age, respectively. All surgical procedures were performed under isoflurane anesthesia (1–5%) and buprenorphine analgesia (0.5–1 mg/kg, *s*.*c*.), and all efforts were made to minimize suffering. After skin incision, the proximal and distal ends of the femoral artery and the proximal profunda femoris artery in the groin were dissected and ligated for both legs. The intervening segments were excised and the dissected sites were sutured with sterile threads. After closing the incision using surgical sutures, the mice were allowed to recover for 7 days. In the sham-operated mice, the same surgical procedure was performed except for the ligation and excision of the artery.

### Treadmill exercise training and tissue sampling

Before FAL surgery, all mice were subjected to a 10-min habituation to a treadmill (Panlab) three times, according to the following protocol: 3 m/min for 1 min and 9 m/min for 4 min, on an incline of 5°. Grouping was performed based on body weight for C57BL/6 mice, and on habituation running time, body weight, and plasma biochemical parameters (glutamic pyruvic transaminase, total cholesterol, triglyceride, glucose, and hemoglobin A1c) for the KK-*A*^*y*^ mice. For exercise training after FAL surgery, mice underwent a total of four to five 30-min treadmill exercise procedures twice a week from 1 week after surgery (exercise groups), according to the following protocol: 3 m/min, 4.2 m/min, 5.4 m/min, 6.6 m/min, and 7.8 m/min for 1 min each; 9 m/min for 5 min; 12 m/min for 10 min; and 15 m/min for 10 min, on an incline of 15°. To monitor the time course of recovery of their running performance, time to exhaustion in each exercise training session was measured. When mice were exhausted, they were allowed to recover (60–90 min), and then returned to the treadmill to achieve a total training time of 30 min. Our preliminary experiments revealed that these repetitive twice-weekly treadmill exercise sessions significantly prolonged time to exhaustion on the treadmill test at 3–4 weeks after FAL surgery in both C57BL/6 and diabetic KK-*A*^*y*^ PAD mice. In the sedentary groups, no mice were subjected to the treadmill exercise until the tissue sampling procedure. For acute assessment, mice were euthanized under pentobarbital anesthesia (50–100 mg/kg, ip) and the right soleus muscles were harvested at the indicated time points (described in the legends of each Fig). For chronic assessment, including individual transcript measurement and RNA seq analysis, the soleus muscles were harvested 4 days after the last exercise. In the present study, we used the soleus muscle, which consists predominantly of slow-twitch fibers, because our preliminary experiments indicated that more genes were significantly altered in the soleus muscle than in the gastrocnemius muscle of these PAD models. The tissues were frozen immediately in liquid nitrogen and stored at −80°C.

### Gene expression analysis

Frozen tissues were dissociated using a GentleMACS dissociator (Miltenyi Biotec, Bergisch Gladbach, Germany) in 1 mL of RLT Plus buffer (Qiagen, Hilden, Germany) containing 0.04 M dithiothreitol. After standing at room temperature for 5 min, samples were centrifuged quickly and then 600 μL of each homogenate was digested using QIAshredder (Qiagen) and centrifuged (20 000 × *g*) for 2 min. The RNA-containing supernatants were purified using an RNeasy Mini kit (Qiagen) and reverse-transcribed using a High-Capacity cDNA Reverse Transcription Kit (Life Technologies, Carlsbad, CA) to obtain cDNA. The mRNA expression levels were then determined by TaqMan quantitative real-time reverse transcription PCR (qRT-PCR; Applied Biosystems, Foster City, CA), using the following predesigned primer/probe sets: *Rplp0*, Mm99999223_gH; *Ppargc1a (Pgc1a)*, Mm01184322_m1; *Il6*, Mm00446190_m1; *Nr4a1*, Mm01300401_m1; *Nr4a2*, Mm00443060_m1; *Nr4a3*, Mm00450074_m1; *Myf5*, Mm00435125_m1; *Myogenin*, Mm00446195_g1; *Tmem8c (Myomaker)*, Mm00481255_m1; *Myh3*, Mm01332463_m1; *Gpr56*, Mm00817704_m1; and *Col3a1*, Mm01254476_m1. Each expression value was normalized to the expression levels of *Rplp0* and calculated relative to the pre-exercise group for the acute phase study and to the sham-operated sedentary group for the chronic phase study.

### RNA sequence analysis

Whole mRNA transcript expression analysis was conducted by Macrogen Inc. (Korea). Briefly, cDNA libraries were constructed using the Truseq RNA Sample Preparation kit (Illumina, San Diego, CA) and paired-end RNA sequencing was performed using the Hiseq2000 system (Illumina). RNA-seq data were processed using Array Studio 8.0.0.78 (OmicSoft Corp., Cary, NC) as follows. FASTQ files were mapped using mouse.B38 as the reference library, and then gene annotations were added to the read count values with ensembl.R78. After checking that the mapping rate was over 80%, the read counts of all samples were exported. Statistical analysis was conducted using the R language (https://www.r-project.org/). To calculate Trimmed Mean of the M-value (TMM) for normalization and to identify mRNAs differentially expressed among soleus muscle from diabetic KK-*A*^*y*^ or C57BL/6 mice, a procedure implemented in the edgeR package was applied. The count per million (cpm) was used in the analysis. Before statistical analysis, for genes with a cpm value greater than 1, at least one sample was retained for further analysis. Up- and downregulated genes were identified as having a *p*-value less than 0.01 and a fold change greater than 1.5. The BaseSpace Correlation Engine software (formally known as NextBio; https://japan.ussc.informatics.illumina.com/c/nextbio.nb; Illumina, Cupertino, CA) was used to conduct Gene Ontology (GO) analysis.

### Measurement of hindlimb blood flow

Mice were kept at 37°C using a BWT-100A animal warming pad (Bioresearch Center, Nagoya, Japan) under isoflurane anesthesia (1–3%). The heart rate was measured by electrocardiography to maintain consistent anesthesia. The hindlimb blood flow was monitored at 8 and 10 min after the onset of warming, using a MoorLDI2-2λsim laser Doppler imaging system (Moor Instruments, Devon, UK). Mice were then allowed to recover under normal conditions. For each measurement time point, the average blood flow perfusion units were calculated for both hindlimbs, and the average was calculated. The average of both time points was used for analysis. All procedures were performed in a blinded fashion.

### Statistical analysis

The results are expressed as the mean ± SD. Statistical significance between two groups was determined by Student’s *t*-test for homogenous data and by Welch’s test for non-homogenous data. Statistical significance between groups of multiple time points was determined by a two-tailed Dunnett’s test. Two-way ANOVA was performed to evaluate statistical differences in the hindlimb blood flow by FAL surgery and by exercise training. Differences were considered significant if *p* < 0.05.

## Results

### Characterization of the two PAD mouse models

**Fig 1:**
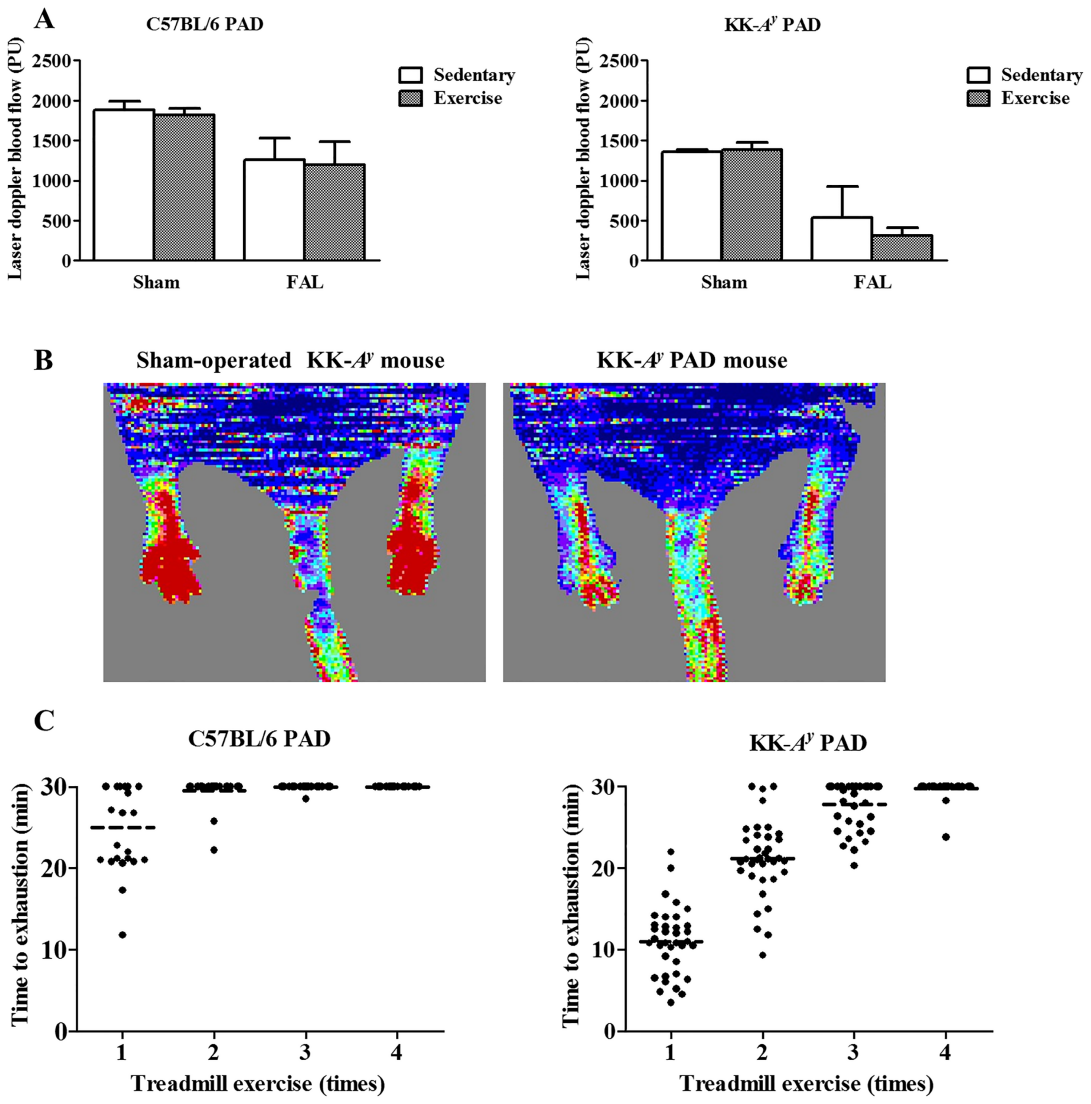
Characterization of the peripheral artery disease (PAD) mouse models. (A) Hindlimb blood flow in sedentary (open bars) and exercised (closed bars) C57BL/6 PAD (left) and KK-*A*^*y*^ PAD (right) mice 3 weeks after femoral artery ligation (FAL). In the exercise groups, mice were subjected to four 30-min treadmill exercise sessions. In the sedentary groups, no mice were subjected to exercise. Two-way ANOVA revealed significant reductions in blood flow after FAL (*p* < 0.01) in both C57BL/6 PAD and KK-*A*^*y*^ PAD mice. Data are expressed as the mean ± SD, n = 4 for sedentary and exercised sham-operated animals, and n = 8 for sedentary and exercised PAD animals. (B) Representative laser Doppler perfusion imaging 3 weeks after FAL surgery. Left, sham-operated sedentary KK-*A*^*y*^ mice; right, sedentary KK-*A*^*y*^ PAD mice. (C) Time course of recovery of the running performance after FAL surgery in exercised C57BL/6 PAD (left) and KK-*A*^*y*^ PAD (right) mice. Dashes represent the mean time to exhaustion for each session in C57BL/6 PAD mice (n = 24) and KK-*A*^*y*^ PAD mice (n = 30).

To characterize the disease phenotypes of normal C57BL/6 and diabetic KK-*A*^*y*^ PAD mice, their hindlimb blood flow was measured by laser Doppler perfusion imaging 3 weeks after FAL surgery (Fig 1A and 1B). A significant reduction in blood flow was confirmed in both PAD models. KK-*A*^*y*^ PAD mice showed a larger blood flow reduction (60–78% reduction *vs*. sham-operated mice) than C57BL/6 PAD mice (33–34% reduction). In addition, exercise training did not affect the blood flow. It should, however, be noted that the comparison of absolute flow units is a limitation of the study design, because laser Doppler flow assessments cannot be compared accurately mouse to mouse; limited effects of exercise training on the hindlimb blood flow should be confirmed using more suitable methods. Fig 1C shows the recovery time-course of the running performance of exercised C57BL/6 PAD and KK-*A*^*y*^ PAD mice after FAL surgery. KK-*A*^*y*^ PAD mice showed delayed recovery compared with C57BL/6 PAD mice. These results suggest a more severe disease phenotype for diabetic KK-*A*^*y*^ PAD mice than for C57BL/6 PAD mice, which is consistent with other studies [18,19].

### Transcripts acutely altered by endurance exercise

**Fig 2:**
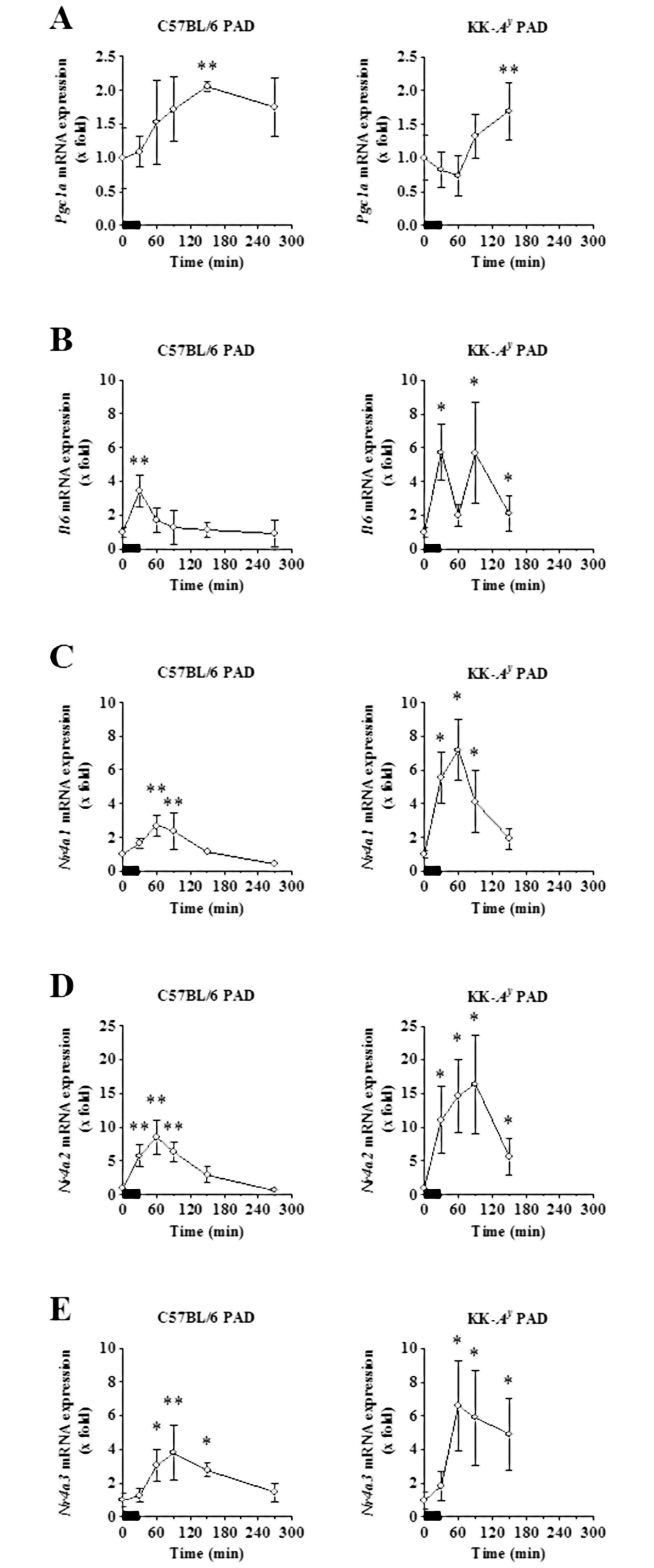
Acute effects of treadmill exercise on the mRNA expression of known exercise-responsive genes. The soleus muscles of exercised C57BL/6 PAD mice (n = 4 per time point, total 24, left panels) and KK-*A*^*y*^ PAD mice (n = 6 per time point, total 30, right panels) were obtained before and after the fifth treadmill exercise session. The mRNA expression levels of *Pgc1a* (A), *Il6* (B), *Nr4a1* (C), *Nr4a2* (D), and *Nr4a3* (E) in the soleus muscles were determined by qRT-PCR, normalized to *Rplp0* expression level, and calculated relative to the pre-exercise group (0 min). The results are expressed as the mean ± SD. *p < 0.05, **p < 0.01 vs. 0 min (Dunnett’s test). Bold bars represent the timing of the fifth treadmill exercise.

Fig 2 shows the acute alterations of known exercise-responsive genes caused by our endurance exercise protocol in the soleus muscles of both C57BL/6 and KK-*A*^*y*^ PAD mice. The mRNA level of *Pgc1a*, a known exercise-responsive gene, increased significantly and gradually after the exercise session in C57BL/6 PAD (2.1-fold at maximum) and KK-*A*^*y*^ PAD mice (1.7-fold). The mRNA level of another known exercise-responsive gene, *Il6*, also increased significantly immediately after the exercise session in both C57BL/6 PAD (3.4-fold) and KK-*A*^*y*^ PAD mice (5.7-fold). In addition, transcript levels of *Nr4a1*, *Nr4a2*, and *Nr4a3* increased after endurance exercise and then gradually decreased in C57BL/6 PAD mice, peaking at 60–90 minutes after the onset of exercise (2.7, 8.5, and 3.8-fold, respectively; Fig 2C–2E). KK-*A*^*y*^ PAD mice also showed upregulation of *Nr4a1*, *Nr4a2*, and *Nr4a3*, with approximately 2-fold greater responses than C57BL/6 mice (7.2, 16, and 6.6-fold, respectively).

**Fig 3:**
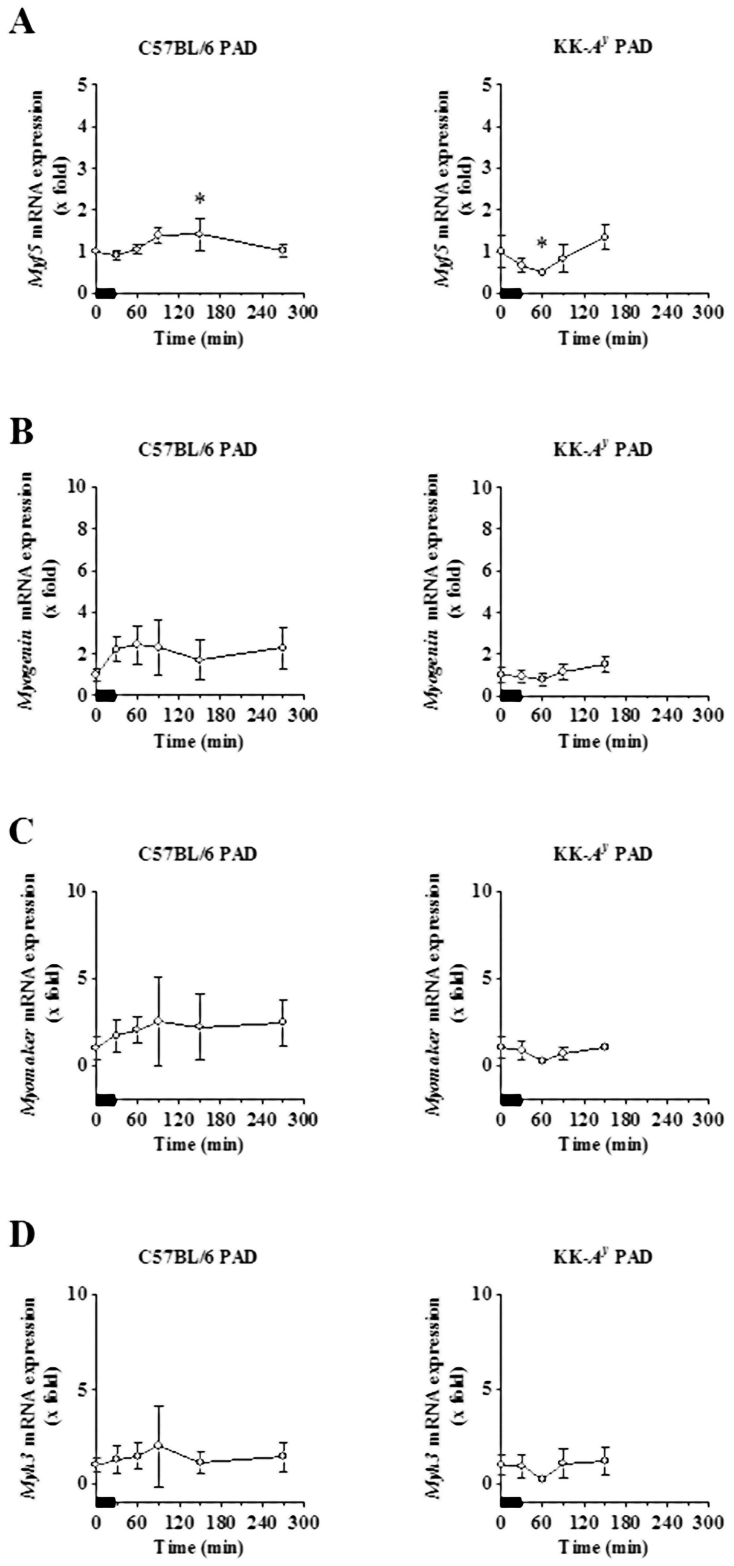
Acute effects of treadmill exercise on the mRNA expression of myogenic and muscle regeneration-related genes. The soleus muscles of exercised C57BL/6 PAD mice (left) and KK-*A*^*y*^ PAD mice (right) were obtained and the mRNA expression levels of *Myf5* (A), *Myogenin* (B), *Myomaker* (C), and *Myh3* (D) in the soleus muscles were determined, normalized, and calculated as described in the legend of Fig 2. The results are expressed as the mean ± SD. Bold bars represent the timing of the fifth treadmill exercise.

Fig 3 indicates the transcript levels of *Myf5*, *Myogenin*, *Myomaker* and *Myh3*, which are known markers of myogenesis and muscle regeneration, in the soleus muscles of both C57BL/6 and KK-*A*^*y*^ PAD mice up to 4 h after the cessation of the exercise. The mRNA levels of these genes showed little alteration during the observation period.

**Fig 4:**
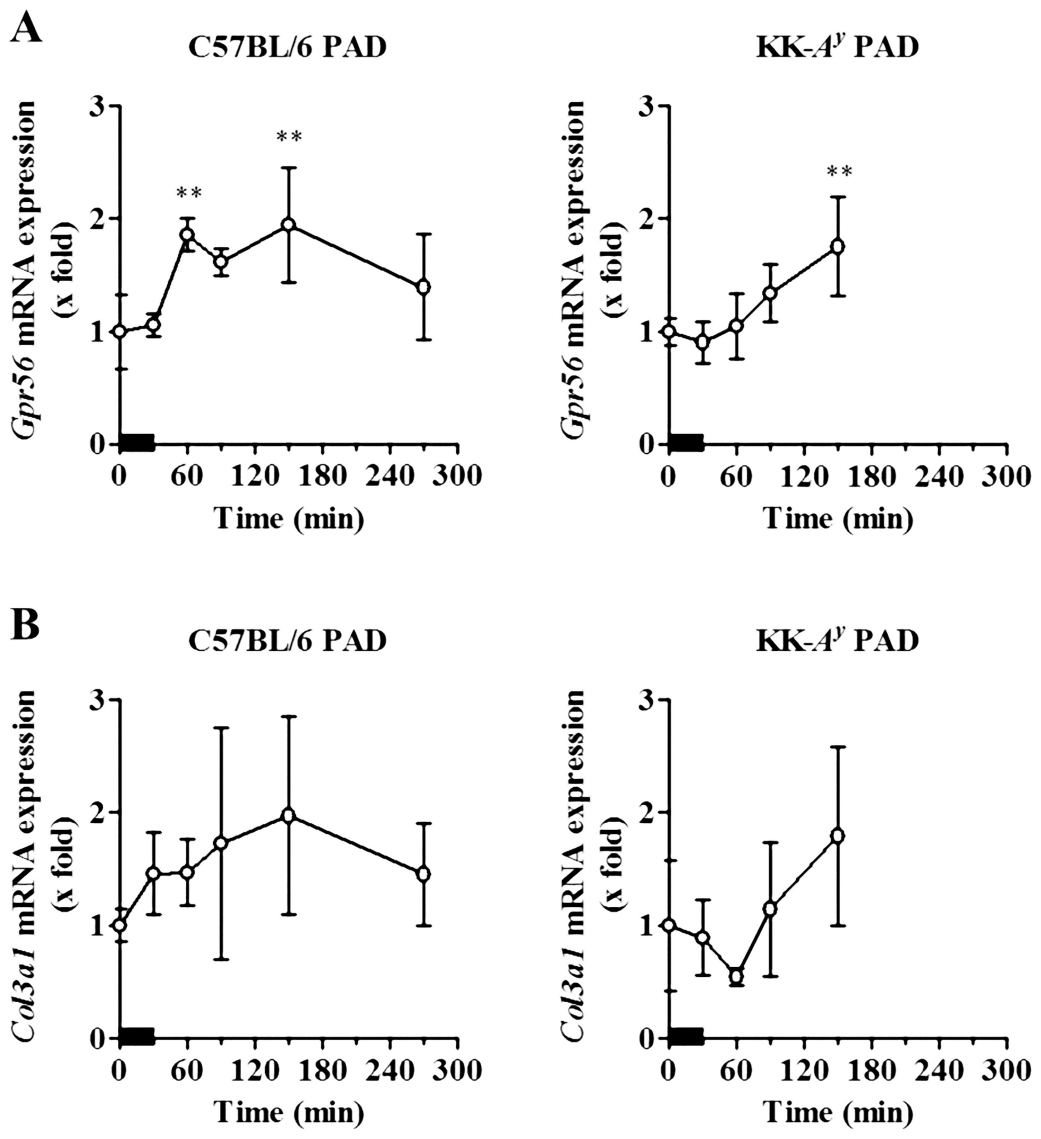
Acute effects of treadmill exercise on the mRNA expression of *Gpr56* and its ligand, *Col3a1*. The soleus muscles of exercised C57BL/6 PAD mice (left) and KK-*A*^*y*^ PAD mice (right) were obtained and the mRNA expression levels of *Gpr56* (A) and *Col3a1* (B) in the soleus muscles were determined, normalized, and calculated as described in the legend of Fig 2. The results are expressed as the mean ± SD. **p < 0.01 *vs*. 0 min (Dunnett’s test). Bold bars represent timing of the fifth treadmill exercise.

The mRNA levels of *Gpr56* and its endogenous ligand, *Col3a1*, were determined using the same samples. *Gpr56* transcripts increased immediately and significantly after the exercise session (Fig 4A) in both C57BL/6 PAD (1.9-fold) and KK-*A*^*y*^ PAD mice (1.8-fold). Although the *Col3a1* transcript level showed an increasing trend in both models, the alteration did not reach statistical significance (Fig 4B).

In addition, no exercise-induced changes were observed in the transcript levels of angiogenesis-related genes such as *Vegfa*, *Des*, and *Cx37* in both C57BL/6 PAD mice and KK-*A*^*y*^ PAD mice (data not shown).

### Transcripts chronically altered by endurance exercise

Tables 1 and 2 summarize the transcript levels of several genes in the soleus muscles of sham-operated sedentary mice (sham sedentary), sedentary PAD mice (FAL sedentary), and chronically twice-weekly exercised PAD mice (FAL exercise), 3 weeks after FAL surgery.

**Table 1:** Chronic effects of treadmill exercise on the mRNA expression in the soleus muscle of C57BL/6 PAD mice.

Gene	Sedentary sham-operated	Sedentary PAD	Exercised PAD
*Pgc1a*	1.00 ± 0.38	2.87 ± 0.64††	2.26 ± 1.03
*Il6*	1.00 ± 0.54	1.41 ± 0.35	1.26 ± 0.38
*Nr4a1*	1.00 ± 0.56	0.66 ± 0.56	0.80 ± 0.11
*Nr4a2*	1.00 ± 0.37	1.71 ± 0.95	1.61 ± 0.39
*Nr4a3*	1.00 ± 1.07	1.16 ± 0.21	0.85 ± 0.33
*Myf5*	1.00 ± 0.15	1.79 ± 0.20††	1.46 ± 0.11‡
*Myogenin*	1.00 ± 0.35	3.18 ± 0.83††	1.63 ± 0.50‡
*Myomaker*	1.00 ± 1.08	11.7 ± 5.18††	5.91 ± 3.86
*Myh3*	1.00 ± 0.42	12.7 ± 3.26††	10.9 ± 4.24
*Gpr56*	1.00 ± 0.22	1.77 ± 0.50†	1.22 ± 0.40
*Col3a1*	1.00 ± 0.02	11.0 ± 1.78††	6.70 ± 0.98‡‡

Results are expressed as the mean ± SD; sedentary sham-operated mice (n = 3), sedentary PAD mice (n = 5), and exercised PAD mice (n = 4)

^†^*p* < 0.05,

^††^*p* < 0.01 *vs*. sedentary sham-operated mice (Student's or Welch's *t*-test).

^‡^*p* < 0.05,

^‡‡^*p* < 0.01 *vs*. sedentary PAD mice (Student's or Welch's *t*-test).

**Table 2:** Chronic effects of treadmill exercise on mRNA expression in the soleus muscle of KK-*A*^*y*^ PAD mice.

Gene	Sedentary sham-operated	Sedentary PAD	Exercised PAD
*Pgc1a*	1.00 ± 0.30	3.30 ± 1.96	2.21 ± 0.74
*Il6*	1.00 ± 0.25	2.27 ± 1.38	1.55 ± 0.48
*Nr4a1*	1.00 ± 0.60	1.29 ± 0.26	1.01 ± 0.23
*Nr4a2*	1.00 ± 0.16	3.74 ± 2.76	2.90 ± 0.97
*Nr4a3*	1.00 ± 0.46	5.92 ± 4.13	4.09 ± 2.05
*Myf5*	1.00 ± 0.09	1.20 ± 0.38	2.31 ± 0.89‡
*Myogenin*	1.00 ± 0.10	2.20 ± 0.84	3.35 ± 1.24
*Myomaker*	1.00 ± 1.23	4.38 ± 5.23	9.81 ± 5.99
*Myh3*	1.00 ± 0.49	4.62 ± 2.08†	8.80 ± 4.40
*Gpr56*	1.00 ± 0.17	2.09 ± 0.56†	1.74 ± 0.21
*Col3a1*	1.00 ± 0.20	11.1 ± 9.44	9.74 ± 5.58

Results are expressed as the mean ± SD; sedentary sham-operated mice (n = 4), sedentary PAD mice (n = 4), and exercised PAD mice (n = 6).

^†^*p* < 0.05 *vs*. sedentary sham-operated mice (Student's or Welch's *t*-test).

^‡^*p* < 0.05 *vs*. sedentary PAD mice (Student's or Welch's *t*-test).

In C57BL/6 sedentary PAD mice, transcript levels of *Pgc1a*, *Gpr56*, *Col3a1*, *Myf5*, *Myogenin*, *Myomaker*, and *Myh3* were upregulated significantly compared to that in sham-operated mice (Table 1). Exercise training inhibited the upregulation of *Col3a1*, *Myf5*, and *Myogenin* significantly. No chronic upregulation by exercise was observed in the transcripts of *Il6*, *Nr4a1*, *Nr4a2*, or *Nr4a*3, which was consistent with the findings of acute and transient induction with rapid recovery after exercise (Fig 2B–2E).

In KK-*A*^*y*^ PAD mice, no significant upregulation was observed in *Myf5*, *Myogenin*, or *Myomaker* mRNA levels compared with those in sham-operated mice, whereas the *Gpr56* and *Myh3* mRNA levels increased significantly (Table 2). Exercise training exerted little effect on the mRNA levels of the genes investigated; only *Myf5* mRNA was upregulated significantly by exercise in KK-*A*^*y*^ PAD mice (Table 2).

To identify differentially expressed genes between sedentary and exercised PAD mice, RNA sequence analysis was also conducted using the same sedentary and exercised PAD mouse samples (Tables 3–6). RNA sequence analysis revealed 33 upregulated and 363 downregulated genes in the soleus muscles of exercised C57BL/6 PAD mice (Tables 3 and 4). In KK-*A*^*y*^ PAD mice, 166 genes were upregulated and 99 genes were downregulated by the exercise training (Tables 5 and 6). Among these differentially expressed genes, only 37 genes overlapped between C57BL/6 PAD mice and KK-*A*^*y*^ PAD mice. Moreover, most (31 of 37) of these overlapped genes were downregulated in C57BL/6 PAD mice, but upregulated in KK-*A*^*y*^ PAD mice, showing a negative correlation.

**Table 3:** List of differentially upregulated genes in the soleus muscle of exercised C57BL/6 PAD mice.

Gene Symbol	FC	Gene Symbol	FC	Gene Symbol	FC
*Irs2*	2.68	*Kcnj2*	1.66	*Vgll2*	1.59
*Hsbp1l1*	2.19	*Slc25a33*	1.66	*Rcan1*	1.57
*Otud1*	1.80	*Acot2*	1.65	*Ptpn3*	1.56
*4632428C04Rik*	1.79	*Rbp7*	1.65	*Mlycd*	1.56
*Abra*	1.79	*Fam134b*	1.64	*Mreg*	1.55
*Slc25a34*	1.79	*Ier3*	1.64	*Ramp1*	1.54
*Nuak1*	1.74	*Klf15*	1.63	*Thrb*	1.53
*Zbtb10*	1.73	*6230400D17Rik*	1.62	*Pde4d*	1.53
*Sh3rf2*	1.71	*Klhl34*	1.61	*Nfil3*	1.53
*C1qtnf4*	1.70	*Klf4*	1.61	*Lmod2*	1.51
*Hspa1a*	1.68	*Nr4a1*	1.59	*Dnajb5*	1.50

FC, fold change.

Sedentary PAD mice (n = 4) and exercised PAD mice (n = 4). Differentially upregulated genes met an average FC criterion of >1.5.

**Table 4:** List of differentially downregulated genes in the soleus muscle of exercised C57BL/6 PAD mice.

Gene Symbol	FC	Gene Symbol	FC	Gene Symbol	FC
*Chga*	-41.09	*Ror2*	-2.80	*Gdf11*	-1.97
*Wnt16*	-29.94	*Fam83g*	-2.79	*Sulf1*	-1.97
*Dlk1*	-26.98	*Cd109*	-2.79	*Adcy7*	-1.96
*Tnn*	-24.17	*Cpxm1*	-2.78	*2610203C20Rik*	-1.96
*Rspo2*	-23.43	*Sntb1*	-2.77	*Has2*	-1.95
*Prr32*	-16.48	*Prc1*	-2.76	*Sytl2*	-1.95
*Rtl1*	-15.48	*Enpp1*	-2.74	*Vgll3*	-1.94
*Lgr5*	-15.05	*Col6a3*	-2.73	*Scd2*	-1.93
*Arhgap36*	-14.34	*Slc37a2*	-2.67	*Il1rl2*	-1.93
*Krt8*	-14.31	*Rbp4*	-2.67	*BC023105*	-1.93
*Mmp12*	-12.99	*Avpr1a*	-2.67	*Pthlh*	-1.92
*Dcx*	-12.88	*Csrp2*	-2.67	*Fam114a1*	-1.92
*Cldn2*	-12.21	*Ccl21a*	-2.66	*Rbp1*	-1.91
*Krt18*	-11.56	*Dkk3*	-2.62	*Klhl13*	-1.91
*Lrrc15*	-11.08	*Pcdhgb1*	-2.62	*Myo5a*	-1.91
*Mest*	-10.79	*Peg3*	-2.61	*Arnt2*	-1.91
*C1qtnf3*	-10.05	*Ephb3*	-2.60	*Itga11*	-1.90
*Nrk*	-10.04	*Adamts20*	-2.60	*Mfap5*	-1.90
*Mirg*	-9.65	*Rnasel*	-2.60	*Frzb*	-1.90
*Rian*	-9.25	*Il20rb*	-2.59	*Rab31*	-1.90
*Meg3*	-8.99	*Basp1*	-2.59	*Col24a1*	-1.90
*Dsp*	-8.96	*Mfsd7a*	-2.58	*Rbms3*	-1.90
*Ptn*	-8.85	*Gldn*	-2.58	*Cdkn1a*	-1.89
*Ripply1*	-8.82	*E2f1*	-2.56	*Tfpi*	-1.89
*AW551984*	-7.95	*Cercam*	-2.54	*Bex1*	-1.89
*Myh4*	-7.67	*Tnfrsf11a*	-2.54	*Sorbs2*	-1.86
*Actc1*	-7.54	*Gamt*	-2.53	*Mtcp1*	-1.86
*Tro*	-7.47	*Ptprf*	-2.53	*Igtp*	-1.85
*Myl4*	-7.16	*Mdga1*	-2.52	*Mrc1*	-1.85
*Peg10*	-7.01	*Itga9*	-2.51	*Boc*	-1.84
*Atp6v0d2*	-6.96	*Thbs2*	-2.50	*Mir99ahg*	-1.84
*Fbn2*	-6.82	*Prkag3*	-2.50	*Npas2*	-1.84
*Postn*	-6.60	*Nr5a2*	-2.47	*Sfn*	-1.84
*Slc6a19*	-6.55	*Ccdc141*	-2.47	*Serf1*	-1.84
*Th*	-6.51	*Efs*	-2.45	*Gbp5*	-1.84
*Msln*	-6.23	*Kcnc4*	-2.45	*Gpc3*	-1.84
*Mybph*	-6.21	*Tdrd9*	-2.43	*Olfr558*	-1.83
*Spon1*	-5.74	*Bid*	-2.42	*Arhgap22*	-1.83
*Myh3*	-5.67	*Serpinb6b*	-2.41	*Dlg4*	-1.83
*Slfn4*	-5.34	*Vcan*	-2.40	*Lepre1*	-1.82
*Aldh3a1*	-5.25	*Adamts7*	-2.40	*Arhgap32*	-1.81
*Plagl1*	-5.09	*Fndc1*	-2.39	*Sh3tc1*	-1.81
*Fjx1*	-4.99	*Srpx2*	-2.37	*Armcx6*	-1.80
*Klf14*	-4.92	*Igf1*	-2.37	*Selp*	-1.80
*Cthrc1*	-4.89	*Atp1b2*	-2.36	*Plat*	-1.80
*Gdnf*	-4.83	*Gatm*	-2.36	*Dupd1*	-1.80
*Grem2*	-4.78	*Eda2r*	-2.36	*Tnfaip3*	-1.79
*Glipr1*	-4.64	*Usp18*	-2.36	*Klhdc8b*	-1.78
*Gpr65*	-4.59	*Tubb6*	-2.35	*Bicc1*	-1.78
*Zfp365*	-4.38	*Ddah1*	-2.35	*Rap2b*	-1.77
*Tnnt2*	-4.34	*Orai2*	-2.34	*Irf8*	-1.77
*6330403K07Rik*	-4.33	*Pi15*	-2.33	*Klf10*	-1.77
*Sprn*	-4.26	*Tnfrsf23*	-2.33	*Mustn1*	-1.76
*Adamts16*	-4.26	*Emilin1*	-2.33	*Vav3*	-1.76
*Igfn1*	-4.22	*Chst2*	-2.33	*Birc3*	-1.76
*Lair1*	-4.22	*Itga4*	-2.32	*9430073C21Rik*	-1.76
*Tnc*	-4.19	*Nhs*	-2.32	*Nabp1*	-1.75
*Cemip*	-4.06	*Fbln7*	-2.30	*Morc4*	-1.75
*Begain*	-3.97	*Kcnq1ot1*	-2.30	*Rcn1*	-1.75
*Ddc*	-3.95	*Fam109b*	-2.30	*Sh3bp1*	-1.75
*Pmp2*	-3.90	*Parm1*	-2.29	*Zfp948*	-1.75
*A930003A15Rik*	-3.89	*Anxa8*	-2.28	*Ttyh2*	-1.74
*Scn5a*	-3.88	*Lhfpl2*	-2.28	*Zc2hc1a*	-1.74
*Oasl1*	-3.87	*Rtp4*	-2.27	*4933439C10Rik*	-1.74
*Prnd*	-3.81	*Sulf2*	-2.26	*Gpx7*	-1.73
*Col12a1*	-3.76	*Ccbe1*	-2.26	*Efemp2*	-1.72
*Kcnk1*	-3.74	*Lyve1*	-2.26	*Fkbp14*	-1.72
*Slfn8*	-3.71	*Adamts3*	-2.25	*Pycr1*	-1.71
*Fam65c*	-3.69	*Ogn*	-2.24	*G0s2*	-1.71
*Mss51*	-3.68	*Osr2*	-2.24	*Rsad2*	-1.71
*Ankrd1*	-3.64	*Lsp1*	-2.24	*Stk17b*	-1.71
*Ifit3*	-3.54	*Primpol*	-2.22	*Junb*	-1.70
*Xlr3b*	-3.53	*Vash2*	-2.22	*Malat1*	-1.70
*Dnm3os*	-3.51	*Traf4*	-2.21	*Htra1*	-1.69
*Map3k7cl*	-3.50	*Fat1*	-2.21	*Camk2d*	-1.69
*Ifit1*	-3.45	*A930004D18Rik*	-2.21	*Mob3c*	-1.69
*Apol9b*	-3.42	*Igsf3*	-2.20	*Sdc2*	-1.68
*Fibin*	-3.42	*Mndal*	-2.20	*Man1a*	-1.68
*Nov*	-3.41	*Msc*	-2.17	*Igsf1*	-1.68
*Tceal7*	-3.39	*Ankrd35*	-2.17	*Angptl2*	-1.66
*Piezo2*	-3.37	*Igfbp4*	-2.16	*Col5a3*	-1.66
*Zim1*	-3.36	*Trp63*	-2.16	*Atp10a*	-1.66
*Ccna2*	-3.33	*Plekha7*	-2.16	*Rin2*	-1.66
*Cpne2*	-3.31	*Gpr64*	-2.16	*Numbl*	-1.66
*Lox*	-3.31	*Rgs10*	-2.15	*Daam2*	-1.65
*Fcgr1*	-3.30	*Elovl6*	-2.14	*Tnfsf10*	-1.65
*Dclk1*	-3.30	*Rcor2*	-2.14	*Chst12*	-1.65
*Cx3cr1*	-3.29	*Tnfrsf22*	-2.14	*Nes*	-1.65
*Pvalb*	-3.27	*Ckap4*	-2.14	*Dab2*	-1.64
*Pamr1*	-3.19	*Fap*	-2.14	*Ext1*	-1.63
*Actn3*	-3.17	*Matn2*	-2.12	*Mdm4*	-1.63
*Gadd45a*	-3.16	*Cd44*	-2.12	*Kdelc2*	-1.62
*Plod2*	-3.12	*Pdk3*	-2.11	*Chpf2*	-1.62
*Rec8*	-3.10	*Mrc2*	-2.10	*Tspan9*	-1.62
*Capn6*	-3.09	*Galnt16*	-2.10	*Ccdc102a*	-1.62
*Aspn*	-3.08	*Mybpc2*	-2.10	*Scai*	-1.61
*Zap70*	-3.07	*Tas1r1*	-2.10	*AI480526*	-1.60
*Hmcn1*	-3.06	*Cdr2l*	-2.09	*Sesn2*	-1.59
*I830012O16Rik*	-3.06	*Entpd7*	-2.09	*Gprc5c*	-1.59
*Oasl2*	-3.02	*Slc25a24*	-2.08	*Fam65b*	-1.59
*Oas1a*	-3.01	*Zfp760*	-2.08	*Fndc3b*	-1.59
*Col5a2*	-3.00	*Tmsb10*	-2.07	*Atp13a3*	-1.59
*Ptch2*	-3.00	*Sertad4*	-2.07	*Zfp503*	-1.57
*Col7a1*	-2.97	*Abi3bp*	-2.06	*C130074G19Rik*	-1.56
*Alx4*	-2.96	*Ifi203*	-2.06	*Aebp1*	-1.56
*Myog*	-2.96	*Fbxl22*	-2.06	*Tanc2*	-1.56
*Lrrc55*	-2.96	*Loxl1*	-2.04	*Sort1*	-1.56
*C1ql3*	-2.96	*Myod1*	-2.04	*Ift122*	-1.55
*Sfrp2*	-2.95	*Spred3*	-2.04	*Ppp1r3d*	-1.55
*Mex3a*	-2.93	*Dse*	-2.03	*Fmr1*	-1.55
*Cacnb3*	-2.92	*S100a4*	-2.02	*Iffo1*	-1.55
*Mmp3*	-2.92	*Cdkn1c*	-2.01	*Ctsz*	-1.54
*Ryr3*	-2.92	*Lgals3bp*	-2.01	*Pear1*	-1.52
*Mstn*	-2.91	*Atp1b4*	-2.01	*Ttc14*	-1.52
*Mmp23*	-2.90	*Zfp354c*	-2.00	*Mid1*	-1.52
*Gxylt2*	-2.90	*Mafa*	-2.00	*Tmem44*	-1.52
*Wisp1*	-2.89	*Adam19*	-2.00	*Mtap*	-1.52
*Marcksl1*	-2.88	*Gk5*	-2.00	*Lgals1*	-1.52
*Trim46*	-2.87	*Bbc3*	-1.99	*Txndc5*	-1.51
*D430019H16Rik*	-2.85	*Hps1*	-1.98	*Dusp6*	-1.51
*Foxd3*	-2.82	*Pcdh18*	-1.97	*Creb3l2*	-1.50

FC, fold change.

Sedentary PAD mice (n = 4) and exercised PAD mice (n = 4). Differentially downregulated genes met an average FC criterion of <-1.5.

**Table 5:** List of differentially upregulated genes in the soleus muscle of exercised KK-*A*^*y*^ PAD mice.

Gene Symbol	FC	Gene Symbol	FC	Gene Symbol	FC
*Tnn*	47.79	*Sbk3*	2.14	*Fbln7*	1.66
*Dhrs9*	9.29	*Mboat2*	2.12	*Tlr2*	1.65
*Chrna9*	7.20	*E2f2*	2.12	*Agbl1*	1.65
*Tmem8c*	6.75	*Bex1*	2.10	*Map1b*	1.64
*Kcnf1*	5.25	*Myh4*	2.09	*Tfcp2l1*	1.64
*4930539E08Rik*	4.89	*Kcnk5*	2.08	*Itga9*	1.64
*Igf2*	4.58	*Vash2*	2.06	*Lrch1*	1.64
*Neu2*	4.45	*Baiap2l1*	2.04	*Soga1*	1.62
*Zfp385c*	4.37	*Myom2*	2.04	*Lnx2*	1.62
*Slc38a1*	4.25	*Malat1*	2.03	*Clcf1*	1.62
*Myh8*	3.97	*Ptgfr*	2.02	*Ldlrad3*	1.61
*Dsp*	3.75	*Mast4*	2.00	*Col20a1*	1.61
*Dhx30*	3.74	*Enox1*	1.96	*Igf2r*	1.60
*Mtus2*	3.67	*Wnt2b*	1.93	*Zc2hc1a*	1.60
*Nxph4*	3.59	*Klf5*	1.93	*Wee1*	1.60
*Sln*	3.50	*Hipk4*	1.92	*Ubash3b*	1.60
*Myh3*	3.42	*Fam129a*	1.92	*Strip2*	1.60
*Slc6a17*	3.39	*Serpinb6a*	1.90	*Cdkn1a*	1.59
*Kcne1l*	3.28	*Dclk1*	1.89	*Gk5*	1.59
*Zdbf2*	3.25	*Phlda1*	1.89	*9330151L19Rik*	1.58
*3632451O06Rik*	3.17	*B930095G15Rik*	1.87	*Agtrap*	1.58
*Serpinb1a*	2.98	*Arhgap28*	1.87	*Cx3cl1*	1.58
*Mamdc2*	2.92	*1110046J04Rik*	1.84	*Macf1*	1.58
*2310015K22Rik*	2.91	*Myoz3*	1.83	*Mtap*	1.57
*Rps6ka6*	2.88	*Ier3*	1.83	*Abca1*	1.57
*Peg3*	2.85	*Gatm*	1.83	*Tceal7*	1.57
*Rrm2*	2.78	*Tnfrsf23*	1.82	*Gnb3*	1.57
*Ahnak2*	2.74	*Pfkfb3*	1.82	*Akr1b8*	1.57
*Zim1*	2.72	*Kbtbd11*	1.81	*Cdc25b*	1.56
*H19*	2.65	*Soat1*	1.81	*Abr*	1.56
*Ngef*	2.59	*Slc22a17*	1.81	*Btnl9*	1.56
*Piezo1*	2.58	*Tmem30b*	1.80	*Dnm3os*	1.56
*Peg10*	2.56	*Mvd*	1.78	*Ociad2*	1.55
*Map3k9*	2.54	*Capn6*	1.77	*Hcn2*	1.54
*Pak1*	2.54	*Sbk2*	1.77	*Angptl2*	1.54
*Gvin1*	2.52	*Sh3rf1*	1.74	*Tubb2b*	1.54
*Ucp2*	2.50	*Fgd3*	1.74	*Col6a3*	1.54
*Grik3*	2.48	*Myl4*	1.74	*Mall*	1.54
*Col7a1*	2.45	*Fst*	1.73	*Sec14l5*	1.54
*Lgals3*	2.45	*Per1*	1.73	*Raph1*	1.54
*Csnk2a1*	2.43	*Hotair*	1.72	*Acer2*	1.54
*Ctxn3*	2.43	*Lrrc74b*	1.71	*Sacs*	1.53
*Cdk6*	2.42	*AI838599*	1.71	*Rfx5*	1.53
*Frrs1*	2.41	*Gcnt1*	1.70	*Itm2a*	1.52
*Maged2*	2.40	*Galnt5*	1.69	*Klrg2*	1.52
*Rin1*	2.30	*Zfp568*	1.69	*Fryl*	1.52
*Ptpn13*	2.21	*Meg3*	1.69	*Cobll1*	1.52
*C77080*	2.21	*Trim47*	1.69	*Tmbim1*	1.52
*Vwa3a*	2.21	*Slc25a24*	1.68	*Zbtb37*	1.51
*Cnr1*	2.18	*Galm*	1.68	*Grip2*	1.51
*Zfp697*	2.18	*Mamstr*	1.68	*Relb*	1.51
*Arhgef37*	2.18	*Calml4*	1.68	*Zfp14*	1.51
*Prune2*	2.18	*Trp63*	1.67	*Cpd*	1.51
*Ky*	2.17	*Npnt*	1.67	*Cadm1*	1.50
*Eps8l2*	2.15	*Nfkb2*	1.67		
*Eda2r*	2.14	*Adam19*	1.66		

FC, fold change.

Sedentary PAD mice (n = 4) and exercised PAD mice (n = 4). Differentially upregulated genes met an average FC criterion of >1.5.

**Table 6:** List of differentially downregulated genes in the soleus muscle of exercised KK-*A*^*y*^ PAD mice.

Gene Symbol	FC	Gene Symbol	FC	Gene Symbol	FC
*Fcgbp*	-68.06	*2310040G24Rik*	-1.98	*Iqcg*	-1.67
*Adamts8*	-5.27	*Cacna2d3*	-1.98	*Cuzd1*	-1.66
*BC048679*	-3.51	*Ppp1r1a*	-1.95	*Tiam1*	-1.66
*Cdh22*	-2.85	*Mpped2*	-1.95	*Dpyd*	-1.64
*9030617O03Rik*	-2.53	*Dupd1*	-1.95	*Rspo3*	-1.64
*Lrtm1*	-2.52	*Nt5c1a*	-1.93	*Ephx2*	-1.63
*A930004D18Rik*	-2.41	*Timp1*	-1.92	*Kif5c*	-1.63
*Crybb1*	-2.41	*Hdhd3*	-1.92	*Krt222*	-1.63
*Amph*	-2.37	*Kcnk2*	-1.91	*Smco1*	-1.63
*Lypd6*	-2.36	*C530008M17Rik*	-1.90	*Fndc5*	-1.62
*Pde3b*	-2.35	*Osbpl6*	-1.89	*Galt*	-1.61
*Mchr1*	-2.35	*Ankrd23*	-1.88	*Mylk2*	-1.61
*Ankrd1*	-2.31	*2310047D07Rik*	-1.84	*St3gal5*	-1.60
*Dusp26*	-2.27	*Fbp2*	-1.82	*Alpl*	-1.60
*Slc15a5*	-2.21	*Mlph*	-1.82	*Insc*	-1.60
*Apobr*	-2.19	*Dhcr24*	-1.81	*Fgf1*	-1.60
*Dkk2*	-2.18	*Dok5*	-1.81	*Lhfpl4*	-1.59
*Kcng4*	-2.16	*A330094K24Rik*	-1.81	*Col22a1*	-1.59
*Cd209f*	-2.16	*Barx2*	-1.76	*Msrb2*	-1.58
*Cda*	-2.15	*Dapp1*	-1.75	*Cox7a1*	-1.58
*Ush1g*	-2.13	*Gins2*	-1.74	*Cux2*	-1.57
*Nog*	-2.11	*Sv2b*	-1.72	*Lyve1*	-1.57
*Tmem132d*	-2.09	*Fam81a*	-1.71	*Hlf*	-1.56
*Rasl10b*	-2.08	*Tmem229b*	-1.71	*mt-Nd4l*	-1.56
*Tbc1d1*	-2.07	*Gsta4*	-1.71	*Aldh1a2*	-1.56
*Gadd45b*	-2.06	*Hes1*	-1.70	*Plin5*	-1.55
*Cds1*	-2.05	*Cytl1*	-1.69	*Rasl2-9*	-1.54
*Retnla*	-2.05	*Wfikkn2*	-1.69	*Tpm3*	-1.53
*Masp1*	-2.04	*Ccrl2*	-1.69	*Sctr*	-1.53
*Kcnc4*	-2.02	*Pde4a*	-1.69	*Nthl1*	-1.52
*Cacna2d2*	-2.01	*Plcd4*	-1.67	*Slc8a1*	-1.52
*Dach2*	-2.00	*Bdh1*	-1.67	*Tst*	-1.51
*Pdzd3*	-1.99	*Lamc3*	-1.67	*Ptgis*	-1.50

FC, fold change.

Sedentary PAD mice (n = 4) and exercised PAD mice (n = 4). Differentially downregulated genes met an average FC criterion of <-1.5.

Gene Ontology analysis was conducted for the differentially expressed genes identified between sedentary and exercised PAD mice (Tables 7 and 8). In the C57BL/6 PAD mice, extracellular matrix-related genes were ranked and downregulated (Table 7). In KK-Ay PAD mice, however, different types of genes, such as channels and transporters, were enriched (Table 8). The directions of the alteration of these enriched genes were different between the two PAD models. The direction of all the top 10 enriched GO terms was downregulation in C57BL/6 PAD mice, while the direction of most (9 of 10) of the top 10 enriched terms was upregulation in KK-Ay PAD mice.

**Table 7:** Enriched Gene Ontology (GO) terms for genes with altered expression induced by chronic treadmill exercise in the soleus muscle of C57BL/6 PAD mice.

Rank	Gene ontology (GO) terms	Direction	p-value
1	Extracellular matrix	down	1.50E-41
2	Proteinaceous extracellular matrix	down	2.20E-39
3	Collagen	down	6.90E-14
4	Ossification	down	9.20E-14
5	Cell migration	down	1.00E-12
6	Muscle contraction	down	1.20E-12
7	Urogenital system development	down	1.50E-12
8	Skeletal system development	down	2.10E-12
9	Contractile fiber	down	4.80E-12
10	Regulation of cell growth	down	5.80E-12

‘down’ indicates downregulation

**Table 8:** Enriched Gene Ontology (GO) terms for genes with altered expression induced by chronic treadmill exercise in the soleus muscle of KK-Ay PAD mice.

Rank	Gene ontology (GO) terms	Direction	p-value
1	Contractile fiber	up	3.20E-08
2	Proteinaceous extracellular matrix	up	2.60E-07
3	Regulation of ion transport	down	3.50E-07
4	Extracellular matrix	up	6.90E-07
5	Actin binding	up	8.80E-07
6	Cell leading edge	up	2.10E-06
7	Aging	up	2.30E-06
8	Cell growth	up	2.30E-06
9	Ion channel activity	up	2.80E-06
10	Passive transmembrane transporter activity	up	4.00E-06

‘up’ indicates upregulation; ‘down’ indicates downregulation

## Discussion

In the present study, we detected significant acute and chronic alterations in the expression levels of several genes in the skeletal muscles of two different mouse models of PAD after exercise, and characterized the expression patterns of each gene. The main findings of this study are as follows: 1) exercise increased the mRNA expression of known exercise-responsive genes acutely and significantly in both C57BL/6 and KK-*A*^*y*^ PAD mice; 2) mRNA expression of skeletal muscle regeneration markers *Myf5* and *Myogenin* was upregulated significantly in C57BL/6 PAD mice compared to that in sham-operated mice at 3 weeks after FAL surgery, and this upregulation was inhibited significantly by exercise training; 3) *Gpr56* mRNA transcription was increased acutely and significantly by exercise in both PAD mouse models; and 4) diabetes did not inhibit the exercise-induced upregulation of *Pgc1a*, *Il6*, *Nr4a1*, *Nr4a2*, *Nr4a3*, or *Gpr56* in KK-*A*^*y*^ PAD mice.

In the present study, exercise induced the mRNA expressions of *Pgc1a*, *Il6*, *Nr4a1*, *Nr4a2*, and *Nr4a3* acutely in both C57BL/6 PAD and KK-*A*^*y*^ PAD mice. Several investigators have reported acute and chronic mRNA expression increases of *PGC1A* [21] and *NR4A* family genes [22] in exercised normal animals and healthy humans. The *PGC1* and *NR4A* families play an essential role in the control of cellular energy metabolic pathways [23,24]; therefore, the exercise-induced upregulation of these nuclear receptors and nuclear receptor coactivators is considered an important molecular mechanism underlying exercise-induced benefits through improved muscle energy metabolism [14]. IL-6 is known as a "myokine", a protein produced and secreted by skeletal muscles to fulfill paracrine or endocrine roles in insulin-sensitizing effects following exercise [25]. IL-6 is also reported to play a role in muscle hypertrophy and regeneration through the activation of muscle satellite cell functions [10, 11, 12]. The present results showing the upregulation of these important exercise-responsive genes suggest that molecular signaling to induce these genes could remain functional in the compromised skeletal muscles of animals and patients with PAD.

In C57BL/6 PAD mice, mRNA transcripts of myogenic and muscle regeneration-related *Myf5*, *Myogenin*, *Myomaker*, and *Myh3* were upregulated significantly at 3 weeks after surgery, compared to that in sham-operated mice (Table 1). This result suggested that active muscle regeneration occurred in this model. In fact, previously, we reported the existence of regenerating myofibers, characterized by a central nucleus location, in the calf muscle of this model [16]. Treadmill exercise did not affect the expression of these myogenic and muscle regeneration-related genes acutely during the observation period (at and up to 4 h after cessation of 30-min exercise, Fig 3), whereas exercise training prevented the upregulation of *Myf5* and *Myogenin* significantly in the chronic phase (Table 1). Yang et al. investigated the time-course activation of selected myogenic (*Mrf4*, *Myf5*, *MyoD*, and *Myogenin*) genes after an acute bout of 30-min treadmill running in the calf muscle of healthy subjects [26]. Similar to the present results, they failed to detect significant differences in *Mrf4*, *Myf5*, and *Myogenin* mRNA levels at 0–24 h post-running. However they noted significant upregulation of *MyoD* mRNA levels (by 8.0-fold) at 8 h post-running. These findings suggest that the optimal selection of time points and genes of interest are important to detect exercise-induced alterations in these myogenic and muscle regeneration-related genes. We speculate that the exercise training-induced prevention of *Myf5* and *Myogenin* mRNA upregulation might reflect exercise-induced acceleration of the muscle regeneration process; however, detailed time-course studies of mRNA expression of the optimally-selected gene set should be performed to confirm this speculation.

The present results support the involvement of GPR56 in exercise-related molecular signaling. Rapid upregulation of *Gpr56* after the exercise session was observed in both C57BL/6 and KK-*A*^*y*^ PAD mice, consistent with the finding that *GPR56* expression was induced in humans by resistance exercise [19]. The mRNA expression level of *Col3a1*, an endogenous ligand of GPR56, also showed an upward trend after the treadmill exercise session. In addition, the time course of exercise-induced *Gpr56* mRNA upregulation was similar to that of *Pgc1a* in both C57BL/6 and KK-*A*^*y*^ PAD mice. This result agreed with the finding that *Gpr56* is a transcriptional target of PGC-1α4, a splicing variant of PGC-1α [19].

Diabetic KK-*A*^*y*^ PAD mice showed exercise-induced acute upregulation of *Pgc1a*, *Il6*, *Nr4a1*, *Nr4a2*, *Nr4a3*, and *Gpr56*, similar to that observed in non-diabetic C57BL/6 PAD mice (Figs 2 and 4). This result suggested that the molecular signaling to induce these genes can function in the diabetic skeletal muscle of this model. In the chronic phase, however, exercise exerted relatively weaker effects on mRNA expression in the KK-*A*^*y*^ PAD mice compared with that in the C57BL/6 PAD mice (Table 2). RNA sequence analysis followed by Gene Ontology (GO) analysis also revealed different characteristics of exercise-related alterations in the chronic phase between KK-*A*^*y*^ PAD mice and C57BL/6 PAD mice (Tables 3–8). Many reports have suggested that diabetic skeletal muscles have characteristics different from those of non-diabetic skeletal muscles, such as impaired metabolism [27], increased oxidative stress [28], chronic low-grade inflammatory profiles [29], and impaired extracellular matrix remodeling [30] and regeneration [31,32]. Further detailed studies are needed to clarify how diabetes (and the different characteristics of diabetic skeletal muscles) can affect exercise-induced transcriptional alterations acutely and chronically.

In the acute experiments, KK-*A*^*y*^ PAD mice showed an apparent bimodal change of *Il6* mRNA upregulation that peaked at 30 and 90 min (Fig 2B, right). However, it should be noted that the biological relevance of this bimodal change should be further confirmed, because of the relatively larger variation of the data at the 90-min time point in the present study.

In conclusion, the present study detected significant alterations in the expression of several genes in the skeletal muscles of two different mouse models of PAD upon exercise, and characterized the expression patterns of each gene. These data provide basic information about exercise-induced transcriptional alterations in the skeletal muscles in mouse PAD models.

## Supporting information

S1 File Data for Fig 1. (XLSX)

S2 File Data for Figs 2–4. (XLSX)

S3 File Data for Tables 1 and 2. (XLSX)

S4 File Data for Tables 3–6. (XLSX)
